# Integrating care for frequent users of emergency departments: implementation evaluation of a brief multi-organizational intensive case management intervention

**DOI:** 10.1186/s12913-016-1407-5

**Published:** 2016-04-27

**Authors:** Deborah Kahan, Molyn Leszcz, Patricia O’Campo, Stephen W. Hwang, Donald A. Wasylenki, Paul Kurdyak, Deborah Wise Harris, Agnes Gozdzik, Vicky Stergiopoulos

**Affiliations:** Department of Psychiatry, University of Toronto, 250 College Street, 8th floor, Toronto, ON M5T 1R8 Canada; Mount Sinai Hospital, 600 University Avenue Room: Ste. 925, Toronto, ON M5G 1X5 Canada; Centre for Research on Inner City Health, Li Ka Shing Knowledge Institute, St. Michael’s Hospital, 30 Bond Street, Toronto, ON M5B 1W8 Canada; Dalla Lana School of Public Health, 6th floor, 155 College Street, Toronto, ON M5T 3M7 Canada; Division of General Internal Medicine, Department of Medicine, University of Toronto, 200 Elizabeth St, Toronto, ON M5G 2C4 Canada; Centre for Addiction and Mental Health, 250 College Street, Toronto, ON M5S 2S1 Canada

**Keywords:** Brief intensive case management, Frequent emergency department users, Mental health and addictions, Transitions of care

## Abstract

**Background:**

Addressing the needs of frequent users of emergency departments (EDs) is a health system priority in many jurisdictions. This study describes stakeholder perspectives on the implementation of a multi-organizational brief intervention designed to support integration and continuity of care for frequent ED users with mental health and addictions problems, focusing on perceived barriers and facilitators to early implementation in a large urban centre.

**Methods:**

Coordinating Access to Care from Hospital Emergency Departments (CATCH-ED) is a brief case management intervention bridging hospital, primary and community care for frequent ED users experiencing mental illness and addictions. To examine barriers and facilitators to early implementation of this multi-organizational intervention, between July and October 2012, 47 stakeholders, including direct service providers, managers and administrators participated in 32 semi-structured qualitative interviews and one focus group exploring their experience with the intervention and factors that helped or hindered successful early implementation. Qualitative data were analyzed using thematic analysis.

**Results:**

Stakeholders valued the intervention and its potential to support continuity of care for this population. Service delivery system factors, including organizational capacity and a history of collaborative relationships across the healthcare continuum, and support system factors, such as training and supervision, emerged as key facilitators of program implementation. Operational challenges included early low program referral rates, management of a multi-organizational initiative, variable adherence to the model among participating organizations, and scant access to specialty psychiatric resources. Factors contributing to these challenges included lack of dedicated staff in the ED and limited local system capacity to support this population, and insufficient training and technical assistance available to participating organizations.

**Conclusions:**

A multi-organizational brief intervention is an acceptable model to support integration of hospital, primary and community care for frequent ED users. The study highlights the importance of early implementation evaluation to identify potential solutions to implementation barriers that may be applicable to many jurisdictions.

## Background

Frequent users of emergency departments (EDs) represent a small subset of the population who often share similar challenges, including homelessness, low income, social isolation, mental illness, substance misuse, and chronic medical co-morbidities [1–5]. While definitions vary, frequent ED users are often identified as those with three to four ED visits per year [2]. This small percentage of users tends to have a disproportionately large impact on the healthcare system. For example, a Canadian study of 14,223 patients found that frequent ED users represented 3.1 % of patients but accounted for 13.8 % of visits and experienced significantly longer ED stays and higher admission rates [6].

Consequently, frequent ED users have become a priority consideration for clinicians and policymakers across many health systems [4, 6]. Some research suggests that poor access to primary care is a contributing factor and that connecting frequent users with accessible and timely primary care and encouraging its utilization could be beneficial in reducing ED visits [2, 7–10]. However, some reports suggest that a majority of frequent users are already high utilizers of primary care [4, 11–14]. Complex medical and non-medical concerns and the perceived “attractiveness” of ED care offer other possible explanations [2].

There are several potential causes underlying frequent ED use. Evidence suggests that case management can bridge institutional and community care and decrease acute care utilization among frequent ED users. A randomized controlled trial of telephone-based case management for frequent ED users resulted in reduced outpatient and ED visits, days in hospital as well as decreased hospital admissions costs [9]. In a recent systematic review on the effectiveness of case management for frequent ED users, 8 of 11 studies reported a reduction in ED use, two showed no significant reduction, and one reported an increase in ED use [3]. The case management literature, while promising, has involved heterogeneous intervention designs, populations, and time frames. There is often limited description of the interventions tested and little attention to whether the interventions were delivered as intended. Furthermore, there is scarce evidence concerning the effectiveness of brief intensive case management interventions (usually of a few months duration) in decreasing ED utilization and improving health outcomes for frequent ED users presenting with mental health and addictions problems. As Kumar and Klein [3] note, this population may require longer and more intensive support to achieve recovery due to the complexity of their needs.

### Rationale and approach

Coordinating Access to Care from Hospital Emergency Departments (CATCH-ED) was launched in 2012 in Toronto, Canada’s largest urban centre, providing brief (approximately fourth to six month) transitional case management to help connect frequent ED users with mental health and addictions problems to primary and specialty care, addictions resources, peer support, counseling, and other community based services as needed. This study aims to examine the acceptability of this multi-organizational brief intervention using stakeholder perspectives, and identify barriers and facilitators of early implementation. Qualitative methods were used to address the following questions:

1. What are stakeholder perspectives on a brief multi-organizational intervention for frequent ED users with mental health and addictions problems?
2. What helps and what hinders the early implementation and delivery of a brief multi- organizational intervention in a large urban centre?

An enhanced understanding of these factors can support program development and implementation in other jurisdictions facing similar challenges.

### Conceptual framework

Fixsen and Blase [15] identified four main intervention implementation stages: *exploration*, *installation*, *initial implementation*, and *full implementation*. This study focused on barriers and facilitators of early implementation. To examine early implementation, we utilized an ecological framework, seeking to understand the connections between individual or program level and environmental or contextual factors influencing the process [16, 17]. This framework highlights two broad dimensions influencing implementation: service delivery system factors and support system factors [16]. Service delivery system factors include organizational capacity, community capacity, and characteristics of the intervention [16, 18]. Organizational capacity includes elements such as work climate, staffing, and collaboration with other agencies [16]. Community capacity includes leadership and participation, skills and resources, social and inter-organizational networks, and community values and power [19]. Characteristics of the intervention refer to compatibility of the intervention with the organizations’ values and priorities and its adaptability to the chosen setting [16]. Support system factors are comprised of technical assistance and training [16, 18]. These factors have been found to both facilitate as well as hinder implementation of recent evidence-based mental health interventions [18, 20–24].

## Methods

### Ethics approval

Research Ethics Board approval was obtained from St. Michael’s Hospital, St. Joseph’s Health Centre, and the Centre for Addiction and Mental Health, all affiliated with the University of Toronto.

### Design

This qualitative evaluation, completed 6 to 9 months after the program was implemented, engaged 47 participants in 32 in-depth semi-structured interviews and a focus group of 16 participants. Direct service providers, program managers, and senior administrators participated in the study to expose a diversity of stakeholder perspectives. Investigator triangulation was used to ensure the quality of this research.

### Study participants and participant recruitment

Participants included all frontline service providers, managers and administrators involved in the planning and implementation of the intervention, with the exception of one administrator, who was unavailable. Participants were recruited by the research team through email and telephone contact from a list of eligible participants obtained through the directors of relevant agencies and hospital departments. All study participants provided written informed consent.

### Intervention

CATCH-ED was informed by the Critical Time Intervention model, a time-limited case management intervention designed to support transitions of care for people with mental illness following discharge from hospitals and other institutions [25]. The intervention was developed and implemented in Toronto, Canada, under the oversight of the Frequent Users Advisory Committee (FUAC) in collaboration with 6 general hospitals and 1 specialty hospital, collectively comprising the Toronto Mental Health and Addictions Acute Care Alliance, 4 primary care centres, and 5 community mental health agencies, providing in-kind expertise and resources. The intervention was initially launched at three partner hospitals in February 2012, assigning a transitional case manager (TCM) to each. Frequent users were defined by having had ≥ 5 visits to any one of the participating ED sites within the last year, at least one of them for a mental health or substance use-related concern, as defined by ICD-10 diagnostic codes. Frequent ED users were identified using either frequent users’ lists or automated flagging systems at participating hospitals. TCMs were trained to engage participants, assess their strengths and unmet needs, provide intensive outreach and support, and connect them to appropriate long-term community-based resources, including primary and psychiatric care over four to six months of follow-up, as needed. Each TCM was supervised by managers in their home agency. Primary care for consumers without family physicians was accessed through participating Community Health Centres (CHCs), designed to deliver comprehensive primary care to disadvantaged populations. Other program activities included crisis intervention, supportive therapy, and assistance in obtaining financial support and housing.

### Data collection

#### Semi-structured interviews and focus group

The Consolidated Framework for Implementation Research [26] informed the development of the interview guides, piloted with 3 service providers prior to becoming finalized. Thirty-two in-depth, semi-structured interviews with service providers, managers and administrators in addition to a focus group with 16 members of the FUAC took place between July and October 2012. Administrator, manager and frontline service provider interviews engaged stakeholders from community mental health organizations, hospitals, CHCs, and other project stakeholders. Members of the FUAC included experts in emergency care and mental health, senior administrators, and representatives of the local health authority. The interviews and focus group lasted between 60 to 90 min and explored stakeholders’ perceptions of program strengths and limitations, barriers and facilitators of early program implementation and perceived project acceptability. Interview topics for frontline service providers additionally explored the service delivery process. All data were collected by 2 trained qualitative researchers using a semi-structured interview guide, were recorded digitally and transcribed verbatim.

### Data analysis

Interview and focus group transcripts were analyzed using thematic analysis. Thematic analysis aims to organize, describe, and interpret a set of data in rich detail. A theme aims to capture something significant in the data relative to the research question, representing a patterned meaning within the data [27]. The transcripts were read multiple times using a *line-by-line* approach to identify key concepts, labeled “codes” [28]. Coding was done using NVivo qualitative data analysis software (QSR International Pty Ltd. Version 10, 2012). The codes were compared within and between transcripts. Two members of the research team coded three interview transcripts independently and compared findings. Once consensus was reached, one member of the research team (D.W.) coded the remainder of the transcripts. Once coding was completed, three members of the research team (V.S., D.W, D.K.) met to analyze and group similar codes into conceptual categories. Similar codes were grouped into themes, supported by direct quotations from the transcripts. These broad categories were then reduced to a small set of overarching themes. We gave priority to themes relevant to the research question based on their primacy and intensity in the interviews. The constant comparative method was used to incorporate new themes that were generated throughout the analytic process [29].

## Results

### Description of participants

In-depth interview participants included transitional case managers (*N* = 4), program coordinators (*N* = 2), a program manager (*N* = 1), a consulting psychiatrist (*N* = 1), community mental health agency leads (*N* = 8), hospital stakeholders (*N* = 10), CHC staff (*N* = 4) and CHC physicians (*N* = 2). One of the 16 focus group participants also participated in a one-on-one interview. Thus, a total of 47 individuals participated in the study (Table 1).

**Table 1:** Study participants

Participant groups	Number of participants (*N*)
I. In-depth interviews	
Direct service providers	
Transitional case managers	4
Program coordinators	2
Project manager	1
Consulting psychiatrist	1
Community Health Centre staff and physicians	6
Community Mental Health Agency leads	8
Hospital stakeholders	10
II. Focus group	
Frequent Users Advisory Committee members	16

Table 2 summarizes the barriers and facilitators to early program implementation, which are described in greater detail below.

**Table 2:** Barriers and facilitators of early implementation of a multi-organizational brief intervention for frequent ED users

Barriers	Facilitators
Poor identification and referral processes	Partnership with local health authority
Incomplete understanding of drivers of ED use	Agency commitment
Decentralized structure	ED presence of case managers
Long wait times for other services	Training and technical assistance
Training and technical assistance	

### Program strengths and factors facilitating program implementation

#### Service delivery system- community capacity factors

*Partnerships with local health authorities* facilitated program implementation in several ways. Firstly, local health authorities sponsored the program and allocated funds for services and program administration. Secondly, stakeholders noted that local health authority participation in planning enhanced the project’s ability to obtain priority access to both primary care and mental health and addictions counseling through participating agencies. Lastly, the planning body’s emphasis on evidence-based interventions was named as a factor attracting agency participation and supporting the research component of the project.

*“The government doesn’t want to spend any money on social services unless it’s proven. …What works? What doesn’t work? And they’re going to fund that.” (Hospital stakeholder, 05)*

#### Service delivery system- organizational capacity factors

*Multiple agency commitment* to the project and the target population facilitated program implementation and in-kind contributions by partner agencies. Agency stakeholders spoke positively about the collaborative nature of the initiative, allowing for a diversity of perspectives, helping inform organizational practices.

*“…there are challenges with multiple organizations but that’s also the beauty of it…And the benefits are you learn different ways of doing things and bring the best of all to the table…” (FUAC member)*

A number of stakeholders thought the intervention offered an opportunity to *integrate hospitals and community agencies,* “bridging” the historical disconnection between sectors, which have different resources, philosophies and models of care.

*“[the hospital was] a little bit of an ivory tower before right, they didn’t talk about their challenges, they didn’t seem to feel that the community had a lot of expertise and so I think this has been kind of an opening … that’s been…a nice change” (Community mental health agency lead, 01)*

Finally, the *partnership with the CHCs* was considered a program strength, the “real gem” of the intervention. CHCs combine primary care services with a wide range of community development and health promotion services and are experienced in providing primary care to individuals with mental illness and addictions.

*“I think it is better equipped than… most healthcare providers just because of the CHC multidisciplinary model… we have so many things under one roof like we have physicians, nursepractitioners, dieticians, social workers, counselors, therapists, harm reduction workers and then patient support or client support workers.”(Direct service provider, 01)*

#### Service delivery system- characteristics of the intervention

Another identified program strength was the *ED presence* of the TCMs, facilitating consumer engagement and prompt response to consumer identified goals.

*“…we need to… create rapport very quickly… And so I’m relatively good at that and so if I get a chance to meet you the likelihood you want to meet with me again increases.” (Direct service provider, 02)*

TCMs also recounted that their on-site presence helped to foster relationships and information sharing with front-line hospital staff.

*“…they recognize me in the hallway and say hello … I’ve had literally phone calls from interns in the emergency saying hey… your guy was here. So I can keep better track now … Gives me collateral information to be better service for people…” ((Direct service provider, 02)*

Furthermore, stakeholders discussed the open-door policy of the program as a strength, as consumer needs fluctuate over time. This *high accessibility* allowed consumers to more easily re-enter the case manager’s roster after discharge, if needed.

*“…if things change and you start to come back to hospital or you feel like you can benefit from the program again it’s just a call away right?…” (Direct service provider, 03)*

*Tailoring of service provision to consumer needs* was yet another program strength and facilitator of implementation, as consumer preference driven services were a priority for the FUAC*. S*ervice providers recognized the need to customize consumer support, matching services to consumer needs and preferences.

*“…you know they look completely different one to the other…needing completely different things… you have to find a way to recognize and respond individually…” (Direct service provider, 04)*

#### Support system - training and technical assistance

TCMs considered their training in the program model to be adequate, although this training was highly variable. Some described having access to additional educational sessions on a variety of relevant topics in addition to regular clinical supervision through their home agency.

*“…[Y]our client is really de-compensating …I always talk to my managers just to let her know like it’ s gotten to that point have I missed any steps, do you have any other recommendations.” (Direct service provider, 03)*

### Program weaknesses and challenges to implementation

#### Service delivery system - community capacity factors

*Lack of accessible community-based services* and lengthy waitlists for community mental health and addiction supports were described as important challenges for this brief intervention. In particular, what was reportedly missing was a*“well-rounded basket of urgent/timely mental health care resources.” (Direct service provider, 01)* CHC services were the only services for which program participants obtained priority access, and some participants saw this as a weakness of the intervention. In particular, the program lacked prompt access to psychiatric care, with CHCs providing occasional one time psychiatric consultations.

*“…We have so many psychiatrists.... Can we not get one or two earmarked for the program?…[the TCM] has to wait in line with everybody else.... There’s no fast track…” (Hospital stakeholder, 05)*

#### Service delivery system- organizational capacity factors

The program experienced low initial referral rates due to *poor identification and referral processes*, including the variable use of frequent user lists and recognition of frequent users by sight. As one hospital informant said *“…might be a good idea to make a list…but more so its word of mouth…oh this person was in last week.” (Hospital stakeholder, 07)*

None of the hospitals had been successful in implementing an automated flagging system during the early implementation stages. Furthermore, each ED had a large and frequently rotating staff, creating challenges to promoting program awareness and consumer referrals. The low profile of the program was due in part to lack of ongoing communication with ED staff after a referral was made.

*“…once we do refer we don’t know what happens. Because the documentation of the case managers belongs to their organization so, we have no idea what has happened or if it's been helpful for the client…” (Hospital stakeholder, 01)*

Additional staffing and structural issues in the ED further impeded the identification and referral of frequent users, including the busy work environment, lengthy referral forms, lack of dedicated ED resources and ED social workers’ hours of service.

*“…a lot of these … come in the evening and they just don’t have the resources because the social workers are usually the referral resources for our ED…[these services are] not available after 5 so, in the evening at night when many of these clients could be coming in they are not being identified or referred.” (Hospital stakeholder, 01)*

As one stakeholder noted, *“…a weakness is that there needs to be people working within the hospital who are strictly focused on working on this project…” (Direct service provider, 05)*

TCMs were not always on site because of daytime office hours and home or community visits to their clients. Another important challenge to program implementation was its *decentralized structure.* Navigating a voluntary partnership with many different organizations was seen by a number of stakeholders as unnecessarily complex. As described above, each agency supervised its own TCM or counselor, leading to unclear overall accountability, and variable adherence to evidence-based practices. The voluntary nature of the partnership led to a certain tentativeness, as one stakeholder put it: *“…you can’t come down too heavy because they could walk away and you* need *them to participate…” (Direct service provider, 06)*

The lack of centralized structure was also a barrier to timely communication among team members. Some TCMs felt isolated in their work and suggested a stronger team structure to facilitate consistent personal and clinical support for them as well as information and resource sharing.

*“We have very little opportunity to talk to each other, to share information with each other, to give each other like tricks, there’s always tricks, right?” (Direct service provider, 02)*

A lack of central clinical leadership was also of concern in the context of the medical and psychiatric complexity of the target population. One stakeholder summed it up:

*“…the challenge has been that the group has had to take what has been offered and try to make a program out of a number of people who work for different organizations, have different you know reporting relationships, have different policies and procedures, have different expectations, different cultures, and not really a sense that working as a team was particularly important.” (Community mental health agency lead, 06)*

#### Service delivery system - characteristics of the intervention

Several stakeholders wondered whether *an incomplete understanding of frequent ED use by TCMs* would be a barrier to the program’s success. For instance, while connecting consumers with primary care (predominantly through CHCs) was an important component of the intervention, lack of access to primary care was not seen as a principal factor underlying frequent ED use. According to service providers, life and illness-related factors and the structure of the local mental health system were often central to ED use. These elements included substandard housing, social isolation, complex mental and physical health needs, and the evening and weekend low barrier access support of the ED. While a number of these elements could be addressed by TCMs, some were addressed less consistently. For example, there was recognition that TCMs may be more effective by providing extended service hours, but this was variably applied across sites. One TCM was available to consumers by phone on a 24/7 basis, while other TCMs were available during business hours from Monday to Friday.

A stakeholder commented:

*“…the services that people need simply aren’t available to them and there needs to be some recognition of that … I will guess that people get into crisis in the middle of the night and they have nowhere else to go and that's going to continue, regardless if you give them 50 doctors… and people are going to get lonely and people are going to have physical or mental health crises or emotional crises and that's going to happen at times when they can’t get access to a doctor or another support…” (Direct service provider, 04)*

#### Support system- training and technical assistance

Finally, inadequate training and technical support, combined with a lack of detailed program standards resulted in *non-uniform service delivery*. TCMs and counselors varied in professional training, experience and access to clinical supervision. In addition, there was a high turnover rate of TCMs, making adequate training more challenging. Since there was no central monitoring and no central support for skills-building and supervision, TCMs and counselors varied in the services provided to consumers. As one stakeholder put it: *“…when we have so manyautonomousorganizations out there working on their own we don’t know what’s happening,there’sa lot of shades of grey, right [?]” (Direct service provider, 07)*

## Discussion

Little is known about how best to integrate hospital, primary and community care for frequent ED users experiencing mental illness and addictions, a population with high rates of medical co-morbidity and social vulnerability [4, 11, 12, 30]. Our findings on stakeholder perspectives of a brief multi-organizational intervention for this population suggest that the approach has several strengths. Furthermore, the multi-level ecological approach used, examining community, organizational capacity, characteristics of the intervention, and support system factors such as training and technical assistance to frontline service providers, was helpful for understanding the early implementation process, a perspective shared by several authors [16, 17, 31].

Collaboration between sectors that do not traditionally work together, coupled with strong endorsement by local health authorities, facilitated commitment by multiple partner agencies and resulted in successful early implementation. Furthermore, joint decision-making by hospital and community agencies enhanced community ownership of the intervention and supported reach and access [16, 32]. The hospital presence of TCMs was a program strength, facilitating consumer engagement, as well as buy-in and information exchange with ED staff. The intervention’s emphasis on individualized care plans, ease of access, and consumer choice enjoyed widespread support among participating agencies. Patient-centered organizations are more likely to effectively implement change or embrace innovation [26, 33, 34], consistent with our findings.

Not surprisingly, certain service delivery and support system factors presented challenges to implementation. One key challenge for this time-limited intervention was the lack of timely access to mental health resources, particularly for psychiatric care and long-term case management. The Canadian literature acknowledges that resource allocation in mental health has not matched the demand for services, resulting in lengthy waitlists [35]. Another challenge was the difficulty integrating frequent user identification and referral processes into the ED setting. Hospitals made inconsistent use of a “frequent users” list, and had no dedicated resources or staff for the target population. Electronic health records can facilitate identification of frequent ED users and support implementation improvements [36–38].

Additional challenges included the program’s decentralized structure, with lack of central supervision, variable quality of supervision, and unclear accountabilities. The implementation literature suggests that decentralization may facilitate initiation of innovation, but centralization facilitates the idea’s implementation [26, 39]. In this case centralization could also alleviate some of the opportunity costs associated with engaging multiple partners both individually and collectively on a regular basis during planning and early implementation, and facilitate program improvements, including training and access to psychiatrists.

In response to these findings, several program improvements were initiated, including: hiring a program manager to provide program cohesion and consistent supervision to TCMs; securing regular consultations with a dedicated psychiatrist; implementing automated electronic flagging systems in the ED; and simplifying the program referral form to facilitate uptake in busy EDs. The program manager and team psychiatrist provided ongoing training in the delivery of evidence-based practices, and consistent supervision to community mental health and CHC frontline staff through regular team meetings. The changes implemented resulted in greater program reach and adherence to program standards, key elements of successful outcomes often overlooked in psychosocial interventions [40, 41].

Further changes may be needed to ensure long-term program sustainability. Alternative models of supporting referral of frequent ED users may be considered, including the presence of a dedicated ED resource. Use of dedicated advanced practice nurses to link older patients in the ED with appropriate community services has been associated with lower hospital admission rates and repeated visits [42].

Finally, this evaluation exposed flaws in our hypothesis that frequent users had difficulty accessing primary care. The evidence that frequent users lack accessible primary care is indeed mixed, with recent studies suggesting that most frequent ED users have primary care physicians and are more likely than occasional ED users to have visited a primary care provider recently [4, 11, 12, 43] implying that frequent ED users may be more likely to access healthcare in general [44]. But while most frequent ED users are connected to primary care, the type of primary care accessed may not adequately meet their needs. Research suggests that CHCs are superior to fee for service or capitation models at providing integrated, comprehensive care to chronically ill populations [45–50] and have lower than expected ED visit rates [51].

A particular strength of this study was the engagement of stakeholders from different sectors in the process of understanding and improving program implementation and refinement. This work nonetheless had several limitations. First, consumer perspectives were not captured during early implementation. Furthermore, the intervention was implemented in a large urban centre with a multitude of services and supports that may not be routinely available in other jurisdictions, limiting generalizability of our findings. Nonetheless, factors facilitating and hindering early implementation of a multi-organization initiative for this population spanning hospital, primary and community care, are likely to be of interest to the many urban centres facing similar challenges.

## Conclusions

This study delineates stakeholder perspectives on a multi-organizational brief intervention bridging hospital, primary and community care for frequent ED users with mental health and addictions challenges. The intervention was generally well accepted by participating organizations. Evaluation has an important role to play in identifying barriers and facilitators to early implementation, and guiding program refinement and full implementation.

### Ethics approval and consent to participate

The study was approved by the research ethics boards of the Centre for Addiction and Mental Health, St. Michael’s Hospital and St. Joseph’s Health Centre, which are affiliated with the University of Toronto and the Toronto Academic Health Science Network. All study participants provided written informed consent.

## Availability of data and materials

Due to the potentially identifying nature of the qualitative data presented in this article, the data supporting the conclusions of this article are not available in a public repository. The data can be available upon request to the authors, in accordance with St. Michael’s Research Ethics Board institutional policies.

## Abbreviations

CATCH-ED: Coordinating Access to Care from Hospital Emergency Departments
CHC: Community Health Centre
ED: Emergency Department
FUAC: Frequent Users Advisory Committee
LHIN: Local Health Integration Network
TCM: transitional case managers
